# Patient-reported outcomes in vaccines research: relevance for decision-making

**DOI:** 10.1080/21645515.2021.1875762

**Published:** 2021-02-19

**Authors:** Desmond Curran, Eliazar Sabater Cabrera, Linda Nelsen

**Affiliations:** a Value Evidence, GSK, Wavre, Belgium; b Value Evidence, GSK, Upper Providence, PA, USA

**Keywords:** Patient-reported outcomes, comparative effectiveness, electronic PRO, health technology assessment, clinical outcomes assessments, health-related quality of life, qualitative research, PRO instruments, public health benefit, vaccines

## Abstract

The development and demand for effective vaccines have witnessed an exponential growth over the last century. In the meantime, the vaccine market involves more knowledgeable stakeholders, with a shift in emphasis by regulatory agencies on understanding the patient perception and experience. The Food and Drug Administration’s publication of the patient-reported outcomes (PRO) guidance has elevated the discipline of PROs and has resulted in a transition from clinician reports of patient outcomes to PROs. This review reports various research methods, which utilize PROs, including qualitative and quantitative research, clinical trials, and patient preference studies. With the advancement of electronic PRO data capture, additional advantages of PROs are being observed and utilized (e.g. as a trigger for clinical endpoints). We discuss uses and advantages of including PROs into the clinical trial program to improve efficiencies, clinical relevance and overall validity of the program in the vaccine field. (See Plain Language Summary)

## PLAIN LANGUAGE SUMMARY

What is the context?

- Potential vaccine recipients want to understand the benefits and risks of a vaccine directly from the patient perspective. Well-defined Patient-reported outcomes (PROs) provide this perspective.
- The Food and Drug Administration (FDA) bases their approvals for interventions on how the patients feel, function and survive, with PROs providing important quantitative estimates of how patients feel and function.

What is new?

- The FDA and European Medicines Agency (EMA) have developed frameworks that ensure that patients’ experiences, perspectives, needs, and priorities are captured and meaningfully incorporated into drug/vaccine development and evaluation.
- This has led to an increased interest in PRO evaluations by other stakeholders including regulatory authorities, ministry of health, health technology assessment bodies, national immunization technical advisory groups (NITAGs), payer groups, key opinion leaders, healthcare providers and potential vaccine recipients.
- The availability of new technologies (e.g. smartphones) has increased the role of virtual observational and clinical studies, using mobile clinical trial platforms assessing PROs with no in-person site visits required.

What is the impact?

- PROs may be incorporated into the research and development program of a vaccine using virtual technology, resulting in a more representative sample that is easier to recruit and retain. This introduces efficiencies and improves the clinical relevance and validity of the clinical trial program.
- The outputs of studies involving PROs are important to communicate the value of vaccination from a patient perspective.
- PRO data may also be included as inputs in public health and cost-effectiveness models, to further inform decision-makers on the value of vaccination.

## Introduction

Over the last century, there has been an exponential growth in the development of effective vaccines to prevent infections, providing substantial benefits to society.^1^ The increasing demand for vaccines is due to factors such as identification of new pathogens, ongoing global immunization campaigns, increased use of vaccines beyond the pediatric segment, notably in older adults and pregnant women, and development of therapeutic vaccines.^1,2^ In the meantime, the vaccine market encompasses more knowledgeable customers, growing cost pressure from payers, and further need for asset differentiation in a very competitive market, i.e. going beyond traditional endpoints of disease prevention.^3^ Potential vaccine recipients want to understand the benefits and risks of a vaccine directly from the patient perspective, driving the increased focus on patient-reported outcomes (PROs). As experts on what it is like to live with their condition, patients are uniquely positioned to inform this context for vaccine development and evaluation. In many countries, patient views are now included as part of the HTA (Health Technology Assessment) processes.^4^

A prophylactic or preventative vaccine involves introducing antigens into an otherwise “healthy” person’s body.^5^ The goal is to induce the individual’s immune system to produce an immune response against those foreign antigens thereby becoming immune to the associated illness, and as such, preventing infection/disease (e.g. rotavirus gastroenteritis, respiratory syncytial virus, and invasive meningococcal disease). Therefore, it is important to understand the impact of disease development and disease prevention on how a patient feels or functions from a patient/vaccine recipient perspective. It is worth noting, however, that prophylactic vaccines are not guaranteed to be 100% effective in preventing disease. Even when a vaccine may not prevent disease it may result in the reduction of disease severity in those individuals who develop disease post-vaccination (e.g. rotavirus, pertussis, influenza, herpes zoster),^6–8^ thereby reducing the impact of disease on how the patient feels or functions.^9,10^ Therapeutic candidate vaccines are meant to help the body defend against an illness that is already present (e.g. herpes simplex virus, chronic hepatitis B), and against health threats including viruses such as oncoviruses.^2^ In such a setting, it will be important to demonstrate that how a patient feels and functions is maintained or improved through vaccination compared to the outcome where individuals are not vaccinated.

PRO instruments are used to measure the experience of disease-related symptoms (e.g. zoster specific pain, using the zoster brief pain inventory [ZBPI]),^11^ disease impact (e.g. sleep or mood disturbance from zoster pain using the ZBPI),^11^ and larger health-related quality of life (HRQL) concepts which may be disease-specific (e.g. cancer specific such as the European Organization for Research and Treatment of Cancer quality of life questionnaire [EORTC QLQ-C30]),^12^ or more general (e.g. Short-Form-36 [SF-36]).^13^

The Food and Drug Administration (FDA) has developed a patient-focused drug development (PFDD) initiative.^14^ This framework is a systematic approach to help ensure that patients’ experiences, perspectives, needs, and priorities are captured and meaningfully incorporated into drug/vaccine development and evaluation. Similarly, the European Medicines Agency (EMA) has developed a framework for interaction between the EMA, patients, consumers and organizations.^15^ FDA approvals are based on demonstrated improvements in how patients feel, function or survive. The FDA defines PRO endpoints as direct assessments of the impact of an intervention on how a patient feels or functions.^16^ Traditional objectives such as pulmonary function, tumor size, or biological markers do not directly assess improvements in how patients feel or function and are consequently defined as indirect assessments. Thus, the FDA requires evidence that the treatment effect for an indirect assessment reflects a treatment effect on how patients survive, feels or functions (direct assessment), to demonstrate evidence of clinical meaningfulness.^16,17^

PROs have served as a primary endpoint in pivotal phase III clinical trials when developing a vaccine, as seen in the Merck Shingles Prevention Study, but have also emerged as important vaccine secondary and/or exploratory endpoints for regulators, HTA bodies, payers, policy experts, healthcare providers and patients.^18,19^

## Patient-reported outcomes (PROs)

PROs are a subset of a larger family of assessments known as clinical outcome assessments (COA) that may be used to understand the broad impact of a new health technology. The four types of COAs are PRO measures, clinician-reported outcome (ClinRO) measures, observer-reported outcome (ObsRO) measures and performance outcome (PerfO) measures.^16,17^ A PRO is a measurement based on a report that comes from the patient or “healthy” individual about the status of their health condition without amendment or interpretation of the report by a clinician or anyone else.^17^ PROs include patient symptom diaries which may assess attributes of duration or severity of symptoms, questions on aspects of function such as physical function or activities of daily living or emotional impacts (e.g. fear of contagion), in addition to multi-item, multi-domain instruments measuring aspects of HRQL. HRQL is a multidimensional measure of the health and treatment experience of the patient, generally involving physical, social, role, and emotional domains. In the US, 75% of PRO label claims were granted for signs and symptoms, 13% for activity limitations and 13% for HRQL. While in the EMA, a higher proportion (31%) of PRO label claims were granted based on HRQL endpoints.^20^

## Robustness of PROs as an endpoint within a clinical trial

Although PROs are sometimes considered to be subjective in nature, it is important to note that prior to inclusion of a PRO instrument into a clinical trial to support endpoints for regulatory claims, the instrument needs to have established criteria for content and construct validity, reliability, responsiveness, interpretation and acceptability.^17^ The instrument also needs to measure a defined concept of patient experience of disease or treatment that is relevant for the target population (content validity). Development of a PRO instrument is a long and complex process involving multiple steps: establishing the concepts to be measured based on qualitative research with patients (content validity); identifying the items which represent the concepts and defining an appropriate response scale and recall period; determining how items should be combined to form domains; appropriate scoring, and assessing psychometric properties including scale validity and measurement precision; and developing guidelines for interpretation. Several guidelines for assessing the properties of PRO instruments have been developed by international societies.^21,22^ In addition, the FDA has published recommendations for establishing content validity and assessing the psychometric properties of PROs.^17^ When instruments that are fit for purpose for the target population are employed together with appropriate statistical methods, consistent and robust results across clinical studies are observed.^9,10,23^

## PRO instrument selection

The choice of PRO instrument will be strongly influenced by the value proposition of the product, which includes statements on the target population, the clinical benefit (e.g. prevention of painful conditions), the health economic benefit (e.g. utility or quality adjusted life year [QALY] gain) and the societal perspective (e.g. promotes healthy aging\HRQL and/or quality of life of a caregiver).

Broadly speaking, disease\symptom-specific instruments may be more influential from a regulatory perspective, utility questionnaires from a recommendation and reimbursement perspective and generic questionnaires from a patient perspective. However, all instruments may influence decisions across different areas of the decision-making process.

## Research methods using PROs

To build credible PRO arguments, one needs data showing that the new intervention has a significant impact on endpoints that are meaningful to healthcare decision-makers, patients and/or society. These endpoints can be disease severity, disease impact, or patient preferences for one health state over the other.

### Qualitative research

The FDA has expanded the role of patient-focused drug development and encourages the collection of patient experience data through qualitative research to augment traditional clinical trials as part of the regulatory submission. Qualitative research provides an opportunity to elicit patient’s voices to better understand the patient experiences including disease symptoms and their related impacts and treatment experience of disease, and support a development of a well-defined PRO strategy. Qualitative research with patients may be performed through focus groups, one-on-one interviews (as seen in concept elicitation studies), or novel methods such as real-world data analysis of social media postings or/and moderated on-line discussions.

To establish content validity to support a PRO, it is fundamental to include the patient, or their caregiver, when the patient is not able to self-report (e.g. young children; adults with dementia), in developing new instruments and when investigating if existing PRO instruments are fit-for-purpose to assess and measure the concepts of interest (e.g. cognitive debriefing) in the target population. In-depth interviews, as opposed to focus groups, allow for more granular individual insights and experiences to be uncovered. The analysis of qualitative research data involves coding and analysis of patient data (e.g. transcripts of interviews) using structured methods to identify and group concepts.

### Quantitative research on disease impact

Quantitative research provides an opportunity to quantify the burden of disease and quality of life impact on a sample of individuals. These data are important in increasing the level of knowledge and awareness about the disease and its complications. PRO instruments measure concepts ranging from discrete symptoms or signs such as cough severity or frequency of cough, to the overall state of a condition, where both specific symptoms and the impact of the condition on function, activities, or feelings can be measured alongside feelings about the condition or treatment/vaccine (e.g. fear of getting infected; infecting others, disease impact; having to avoid certain situations).

The question of what instruments to use should be driven by both qualitative research and literature reviews to ensure that the concepts that are relevant to the patients are appropriately captured using instruments that are fit for purpose in the target population. For example, for herpes zoster, it is important to assess the pain/allodynia/pruritis and the associated impact on activities of daily living, and health-related quality of life. As such, the ZBPI disease-specific instrument and generic PRO tools such as the SF-36 and EQ-5D may be appropriate. However, if existing PRO instruments are not adequate, it may be necessary to either modify an existing instrument or develop a new instrument.

### Clinical trials

When developing the product profile and value proposition, it is important to consider how the vaccine could potentially impact the patient from a patient-centric perspective. Even at this early stage, it is important to consider what wording could be included in the regulatory label to support the product profile, the value proposition and promotional claims (i.e. starting with the end in mind). Concepts should be identified which best illustrate how the disease is altered by the vaccine under development in the study population. It is imperative to understand the impact of disease on PROs (i.e. disease process, signs and symptoms) from previous qualitative and quantitative research and the rationale for any potential vaccine effect on the disease. This may include assessing the overall reduction in disease impact and/or reduction in disease impact in breakthrough subjects.^9^ Based on literature reviews and external opinion, a conceptual framework should be hypothesized to support the measurement of the concept of interest and identify the domains and items to be measured, which must be subsequently validated by patient involvement.^17^ Various PRO instruments may already exist which measure this concept in the disease area of interest and in the relevant population matching the development program.^17^

It is important to define the role of a PRO endpoint in the clinical trial as either a primary, key secondary, or exploratory endpoint. Note that historically for FDA label inclusion, generally only primary or secondary endpoints are considered,^17^ whereas the EMA has considered exploratory endpoints if those show clinically meaningful effects.^15^ The objectives and endpoints developed within the clinical trial should be developed bearing in mind the potential label wording to support promotional claims.^17^ Consequently, the instrument to be used, the frequency of assessment, recall period, definition of the endpoint and statistical methods are all critical elements in assuring that the endpoint can be assessed appropriately.^15,17^ Other important considerations are the patient population, languages, and mode of administration.

### Patient preference studies

A Discrete Choice Experiment (DCE) is a method for eliciting preferences regarding alternative scenarios or options.^24^ Participants are presented with alternative hypothetical scenarios and are asked to indicate their preferred option, with each option involving several attributes (outcomes) with different levels.^24^ DCEs have been commonly used to (1) estimate the difference in utilities between health states; and (2) help to prospectively identify which outcome of a trial is more relevant to the patient.^24^

In the case of vaccines, it may be informative to explore what attributes individuals are willing to trade off to prevent disease and consequently have the peace of mind from protection for themselves and/or potential family members, including through maternal vaccine protection or through herd immunity.

Preference methods to measure utilities also include time-trade-off (TTO) and standard gamble, while the estimation of utilities can also be done using PROs such as the health utilities index (HUI), EQ-5D or SF-36.^25^

### Assessment of PROs

It is important to note that a PRO strategy should be developed and integrated early into the research and development process. For example, it is important to identify the concepts that are relevant for patients involving qualitative interviews. The potential development of an instrument, ensuring that the instrument is fit for purpose in the specific population and in the disease area of interest, takes a considerable amount of time. It is important to perform quantitative research to identify the appropriate schedule of assessments prior to the phase III clinical trial. Additional quantitative research, clinical trials and preference studies may then be performed in parallel.

Note that in prophylactic vaccine studies PRO assessments are frequently completed at baseline, in otherwise “healthy” individuals prior to infection, and subsequently during the episode. PRO assessments can also be done after the initial episode has resolved to explore long-term effects of an episode. Consequently, PRO-studies in vaccine clinical trials are more cumbersome than in treatment trials because the sample size is much larger in vaccine clinical trials. It may also be important to assess disease-specific questionnaires at baseline, as some individuals may already have underlying conditions associated with symptoms that are consistent with infection (e.g. shortness of breath associated with asthma or chronic obstructive pulmonary disease (COPD) which is an important concept for individuals with a respiratory infection).

### Statistical analysis of vaccine studies

Several standards have been developed to harmonize the statistical analysis of PRO data including the more recent Setting International Standards in Analyzing Patient-Reported Outcomes and Quality of Life Endpoints (SISAQOL) guidelines.^26,27^ The guidelines are also relevant for PRO data from vaccine clinical trials, yet they primarily focus on individuals who are ill or have disease. Consequently, endpoints such as the change in relief of symptoms from baseline, time to reduction in disease symptoms and responder analysis are utilized to show the proportion of patients experiencing meaningful improvement.^26,27^ The statistical analysis of PRO data from a prophylactic vaccine trial introduces additional specificities in that “healthy” individuals are vaccinated, who may later develop disease. Prophylactic vaccines can reduce the impact of disease on an individual’s HRQL by preventing disease but can also attenuate disease severity in subjects who develop disease despite receiving vaccination.^9^

In vaccine studies, it is important to compare the PRO data in subjects who developed disease in the unvaccinated and vaccinated groups. For example, in herpes zoster (HZ) studies, it was demonstrated that the vaccine attenuated disease severity in individuals who developed disease manifesting in significantly lower pain scores among patients with confirmed HZ in the vaccinated group.^9,23^ It should however be noted that comparing PRO data between those individuals who have developed disease in the unvaccinated and vaccinated groups is subject to selection bias.^28^ Consequently, a burden-of-illness (BOI) statistic has been developed as a composite endpoint to incorporate disease incidence with disease severity and duration. In the herpes zoster (HZ) studies mentioned above, the severity of illness due to HZ was calculated as the area under the curve of the patient self-assessment of pain related to HZ from day 0 to day 182 of the HZ episode for subjects with HZ and as 0 for subjects without an HZ episode.^9,23^ The BOI was then calculated as the sum of the severity of illness scores in a group divided by the sum of the subject years of follow-up in that group.^28^ Vaccine efficacy was then estimated as the relative reduction in the BOI score in the vaccinated group as compared to the score in the placebo group and calculated as one minus the relative risk (i.e. the BOI score in the vaccinated group divided by the BOI score in the placebo group). The BOI statistic has been used in vaccine decision-making,^29^ although some HTA bodies have questioned the use of composite endpoints in a clinical trial setting.^30^ Nevertheless, the BOI statistic is unbiased and has been demonstrated to be a robust measurement of vaccine benefit.^9,10,18,23,28^ As such, the BOI statistic should be considered as an important efficacy endpoint from a PRO perspective in vaccine clinical trials.

## PRO stakeholders

A PRO strategy must be developed considering the decision-makers of vaccination programs at both a regional and country level. A clear PRO strategy is of prime strategic importance to provide direct evidence of patient benefit which will ultimately contribute to the success of a product as it serves as an important communication of the product’s value proposition, expressed in clear PRO value messages, and as a potential differentiator over a competitor or compared to the existing situation. A PRO strategy should be planned and implemented in the earliest stages of product development. In most countries the decision-makers interested in PROs include:

- Regulatory Authorities,
- The Ministry of Health,
- The Advisory Bodies making recommendations on immunization programs (Health Protection Agencies, HTA, National Immunization Technical Advisory Groups [NITAGs] etc.),
- Payers such as: insurance companies, sickness funds, funding bodies, etc.,
- Key opinion leaders (physicians, health economists, etc.),
- Healthcare providers if they have a choice between different options,
- Vaccine recipients, parents, caregivers and patient advocacy groups.

## Additional uses of PROs

PROs may be used in various ways to enhance research within a development program. Recently, FDA has noted that PRO instruments could be applied to help determine patient´s eligibility inclusion criteria, measure safety and/or effectiveness endpoints, either as a standalone or as a component of a composite endpoint.^31^ Table 1 provides an overview of examples where PROs have been utilized, including as inclusion or exclusion criterion or as a case definition within a clinical trial.^19,32^ When a PRO is assessed electronically (i.e. ePRO) the identification of a case can trigger other events such as a telephone call, site visit or the completion of other assessments to be performed according to the study protocol. Electronic capture of PROs also ensures that the source data are ALCOA compliant (i.e. attributable, legible, contemporaneous, original, and accurate) and ALCOA+ compliant (i.e. Complete, Consistent, Enduring and Available).^34^ The availability of new technologies has increased the role of virtual clinical trials and the use of mobile clinical trial platforms assessing PROs in conjunction with wearable devices to track participants with no in-person site visits required.^34^ Virtual studies have the potential to make clinical studies more inclusive, faster and more cost-effective.^34^ This can be achieved using technology, such as smartphones, that study participants may already have available. Virtual studies bring studies directly to the patients at home, creating a more human approach to clinical research. In doing so, studies may be more representative and easier to recruit as well as help retain subjects in an ongoing trial.^34^

**Table 1.** Additional uses of PROs

Use of PRO	Example	Details
As an inclusion/exclusion criteria^32^	The GOLD initiative for COPD established the GOLD “ABCD” assessment tool to classify COPD patients combining spirometry data, risk of exacerbation and symptoms assessment based on PROs.^33^ The CAT is the PRO instrument used to support the GOLD “ABCD”.	The CAT total score is comprised of eight items assessing cough, phlegm, chest tightness, breathlessness, activities, confidence, sleep and energy. The GOLD classification is widely used to define the study population within COPD trials and as inclusion/exclusion criteria.
In the case definition^19^	An ARI case identification was triggered using the FLU-PRO. If subjects experienced 1 day of any respiratory symptom from a pre-defined list of respiratory symptoms (e.g. runny/stuffy nose, sore throat, earache or pain) they recorded symptoms daily using the Flu-PRO, an existing PRO instrument.	ARI (primary endpoint): detection of RSV in at least 1 respiratory sample at the time of illness plus ≥1 symptom from any 2 of 3 locations (upper respiratory tract, lower respiratory tract, systemic) from the FLU-PRO instrument.
To define severity of cases^18^	HZ patients were classified as developing PHN based on the ZBPI scale, an existing PRO instrument. Suspected HZ cases were asked to complete the ZBPI at home every day from rash onset daily for 28 days, and weekly thereafter until either they had been painfree for 4 consecutive weeks or 90 days had elapsed.	PHN was defined as a ‘worst’ pain score ≥ 3 on the ZBPI scale persisting or appearing 90 days or more after rash onset. The duration of PHN is calculated similarly as the period that pain persists using the worst ZBPI pain score.
Virtual Studies^34^	Patients with CKD were followed up using a PRO-based approach to evaluate the effectiveness of the quality of care, use of resources and patient outcomes associated with CKD. A battery of self-assessment questionnaires including the SF-36 domains, EQ-5D and the BIPQ.	The primary endpoint was defined as non-inferiority in loss of renal function (eGFR) comparing patients in the (1) PRO-based remote follow-up and (2) PRO based telephone consultations versus (3) Usual outpatient follow-up (control group)
Surveillance systems to monitor patients^35^	Memorial Sloan Kettering Cancer Center performed a randomized controlled trial, in patients receiving chemotherapy, comparing a surveillance system reporting 12 common symptoms via tablet computers versus usual care consisting of symptom monitoring at the discretion of clinicians, A web-based interface STAR previously established for patients with cancer with high symptom burdens was used in association with the EuroQol EQ-5D instrument.	For those individuals using the surveillance system, automated alerts for severe or worsening symptoms were e-mailed to their nurses prompting intervention. The PRO intervention was associated with significantly fewer emergency department visits and improved overall survival, as well as improvements in quality of life.
In Frailty assessment^36,40^	A Frailty Index was constructed using an accumulation of deficits approach, incorporating medical comorbidities and PROs. The PRO assessment incorporated a baseline assessment of the SF-36 and EQ-5D instruments.	Study subjects were classified as non-frail; pre-frail; or frail based on the constructed frailty index. Vaccine immunogenicity, efficacy, and safety were evaluated stratified by frailty status.
Public health impact and Cost-Effectiveness modeling^37,38^	A multi-cohort Markov model was used to estimate the potential public health and economic impact of the new RZV in the German population ≥ 60 YOA. A prospective study assessing the impact of HZ and PHN on the quality of life of individuals ≥ 50 YOA in Germany was used to estimate utility inputs using the EQ-5D questionnaire.	The QALY loss associated with a HZ only episode (i.e. no PHN) was estimated to be 0.018 in subjects 50–69 YOA and 0.019 in subjects ≥ 70 YOA, and 0.158 for a HZ episode involving PHN.

ARI, acute respiratory illness; BIPQ, brief illness perception questionnaire; CKD, chronic kidney disease; COPD, chronic obstructive pulmonary disease; CAT, chronic obstructive pulmonary disease assessment test; EQ-5D, EuroQol- 5 Dimension; eGFR, estimated glomerular filtration rate; GOLD, global initiative for chronic obstructive lung disease; HZ, herpes zoster; PRO, patient-reported outcome; PHN, post-herpetic neuralgia; RSV, Respiratory Syncytial Virus; RZV, Recombinant Zoster Vaccine; STAR, symptom tracking and reporting; SF-36, Short Form 36; QALY, quality-adjusted life year; YOA, years of age; ZBPI, zoster brief pain inventory

An additional example of the use of PROs can be seen among frail individuals who are at increased risk of poor clinical outcomes.^39^ Frailty may be influenced by an age-related decline in innate and adaptive humoral and cell-mediated immunity which impairs their ability to resist infection and respond to interventions such as vaccination.^40^ Frailty Indices have been constructed using PROs allowing vaccine efficacy and safety to be evaluated by frailty status.^36^

In addition to the acute period of infection, vaccine-preventable disease may be associated with long-term sequelae in children and young adults,^25^ while infected older adults may not completely recover and return to their pre-disease functional ability, thus loosing independence.^41^ In such cases, PRO data are necessary to describe the long-term impact of an infection and consequently the value of vaccination, e.g. by preventing disease, the functional ability is maintained, thus enabling healthy aging.^36,41^ To estimate the public health benefit and cost-effectiveness of vaccination, it is imperative that robust estimates of both the short- and long-term impacts, including the probability of developing sequelae and the QALY loss, are available.^37,38^

Vaccines may also have negative implications for certain aspects of life deemed important to the individual; vaccine reactogenicity is often a necessary but unwanted implication of a vaccine. As such, it may also be important to address the potential negative effects of a vaccine, including the impact of vaccine reactogenicity on an individual’s functioning, to better inform potential vaccine recipients and consequently to support their decision-making on whether they are willing to receive it or not. Healthcare providers, payers (e.g. due to associated costs) and regulators should also be informed of any negative consequences of vaccination. Both vaccine-specific and generic questionnaires have been used to assess the impact of reactogenicity.^42,43^ The rationale for using generic questionnaires is that they allow a comparison of the impact of vaccine reactogenicity and disease impact on concepts such as pain, physical function, vitality, quality of life, and utility loss.^43^

## Discussion

Traditionally, clinical studies were based on clinician-reported outcomes of patients’ health. However, it is now well recognized that PRO measures should be used when assessing concepts best known by the patient or best measured from the patient’s perspective, without interpretation by clinicians or others.^17^ This transition from clinician reports of patient outcomes to PROs, in conjunction with the FDA’s publication of the PRO Guidance, has transformed the discipline of PRO measurement.

Constructing a comprehensive PRO strategy, following well-documented guidelines for the development and validation of PRO instruments, defining endpoints that are relevant for the patient, while matching the development program, and using appropriate statistical methodology will lead to a more robust outcome while increasing the probability of a successful label claim. Once labeling is achieved, the information in the label can be informative to prescribing clinicians and facilitate raising awareness amongst patients and decision-makers at all levels regarding the value of the vaccine.

PROs may be incorporated into the research and development program of a vaccine in many additional ways. As mentioned above, virtual clinical and epidemiological studies using mobile trial platforms may result in a more representative sample as well as being easier to recruit and retain subjects. In addition, events are triggered (e.g. cases or outcomes) resulting in less cases and events being missed and a more streamlined follow-up of such events and cases. These elements can introduce efficiencies and improve both the clinical relevance and validity of the clinical trial program.^16^ The outputs of studies involving PROs may also be included in public health and cost-effectiveness models, informing decision-makers at all levels on the value of vaccination.

It should be noted that the many uses of PRO data may not materialize if study subjects do not receive adequate training on the value of PROs and how to adequately complete PRO assessments and the requirements of the study protocol. The importance of providing training on the rationale and implementation of PROs to patients and clinical staff to obtain high-quality data should not be underestimated.

As with other study procedures, an assessment needs to be performed to understand the benefits of including PROs into a study. For example, a study of a prophylactic vaccine assessing immunological outcomes where few subjects may develop the disease under investigation may be considered inappropriate or unethical for study participants to complete an assessment, which has limited benefit. It is therefore important that sample size and/or associated power calculations are performed in studies that include PROs to ensure that the sample size is sufficiently large to have a high probability of detecting clinically meaningful differences, in order to obtain reliable results and to be able to convince others of their validity.
